# Thyroid Function among Breastfed Children with Chronically Excessive Iodine Intakes

**DOI:** 10.3390/nu8070398

**Published:** 2016-06-28

**Authors:** Inger Aakre, Tor A. Strand, Trine Bjøro, Ingrid Norheim, Ingrid Barikmo, Susana Ares, Marta Duque Alcorta, Sigrun Henjum

**Affiliations:** 1 Department of Nursing and Health Promotion, Faculty of Health Sciences, Oslo and Akershus University College, 0130 Oslo, Norway; ingrid.barikmo@outlook.com (I.B.); sigrun.henjum@hioa.no (S.H.); 2 Department of Global Public Health and Primary Care, Faculty of Medicine and Dentistry, University of Bergen, 5018 Bergen, Norway; tors@me.com; 3 Research Department, Innlandet Hospital Trust, 2609 Lillehammer, Norway; 4 Department of Medical Biochemistry, Oslo University Hospital, 0379 Oslo, Norway; trine.bjoro@medisin.uio.no; 5 Institute of Clinical Medicine, University of Oslo, 0379 Oslo, Norway; 6 Department of Endocrinology, Morbid Obesity and Preventive Medicine, Oslo University Hospital, 0424 Oslo, Norway; UXINRH@ous-hf.no; 7 Neonatology Unit, University Hospital La Paz, University of Madrid, 28046 Madrid, Spain; susana.ares@salud.madrid.org (S.A.); marta.duqueal@salud.madrid.org (M.D.A.)

**Keywords:** iodine excess, urinary iodine concentration, breast milk iodine, iodine intake, thyroid function tests, hypothyroidism, thyroglobulin

## Abstract

Iodine excess may impair thyroid function and trigger adverse health consequences for children. This study aims to describe iodine status among breastfed infants with high iodine exposure in the Saharawi refugee camps Algeria, and further assess thyroid function and iodine status among the children three years later. In 2010, a cross-sectional study among 111 breastfed children aged 0–6 months was performed (baseline study). In 2013, a second cross-sectional study (follow-up study) was conducted among 289 children; 213 newly selected and 76 children retrieved from baseline. Urinary iodine concentration (UIC) and breast milk iodine concentration (BMIC) were measured at baseline. UIC, thyroid hormones and serum thyroglobulin (Tg) were measured at follow-up. At baseline and follow-up, 88% and 72% had excessive iodine intakes (UIC ≥ 300 µg/L), respectively. At follow-up, 24% had a thyroid hormone disturbance and/or elevated serum Tg, including 9% with subclinical hypothyroidism (SCH), 4% with elevated fT3 and 14% with elevated Tg. Children with SCH had poorer linear growth and were more likely to be underweight than the children without SCH. Excessive iodine intakes and thyroid disturbances were common among children below four years of age in our study. Further, SCH seemed to be associated with poor growth and weight.

## 1. Introduction

Iodine deficiency remains a global health problem, but there has been great progress in eliminating iodine deficiency disorders over the last few decades [1,2]. Based on median urinary iodine concentration (UIC) among school-aged children, the number of countries with adequate iodine intake has increased from 67 to 112 over the past 10 years. In addition, iodine excess has become more prevalent over the past decade mainly because of salt iodization, and, in 2013, 10 countries were classified with excessive iodine intakes (median UIC ≥ 300 µg/L) [3]. Fetuses and newborns are high-risk groups for excessive iodine exposure, since their thyroid gland is immature and has less adaptive abilities for high iodine doses than for adults. This is probably because the thyroids of fetuses and newborns are unable to escape from the acute Wolff-Chaikoff effect [4,5,6,7]. In addition, infants are particularly vulnerable when it comes to iodine excess, since thyroid hormone disturbances may affect their growth and developmental skills [8,9]. For breastfed infants, the mother’s iodine intake is of importance, since iodine is excreted through breastmilk [10,11,12].

Although chronical iodine excess in pediatric populations is an area of limited research, studies have shown both increased thyroid volume [13,14] and thyroid disturbances [15,16] in children exposed to excessive iodine. However, there is limited knowledge concerning the longitudinal effects of chronically excessive iodine exposure in children, particularly at the preschool age.

In the southwest of the Algerian desert near the city of Tindouf, approximately 165,000 refugees are living in the Saharawi refugee camps. People living in these camps are exposed to high iodine from groundwater [17], which is probably responsible for endemic goiter among women and children [18,19] and a high prevalence of thyroid dysfunction among women [20,21]. We conducted two studies in the refugee camps to assess the consequences of chronically high iodine exposure in lactating women and their children: a cross-sectional iodine study in 2010 (baseline study) and a second study in 2013 (follow-up study) [20,21]. The main objective of the present paper is to describe breast milk iodine concentration (BMIC) and the iodine status among breastfed children aged 0–6 months living in an area of high iodine exposure (at baseline) and to describe serum concentration of thyroid hormones, thyroid function, and iodine status among the children at three years of age (at follow-up). Our hypotheses were as follows: the children have (a) excess iodine intake in early infancy (baseline) and early childhood (follow-up); and (b) thyroid hormone disturbances and elevated serum thyroglobulin (Tg) during early childhood (follow-up).

## 2. Materials and Methods

### 2.1. Subjects

**Figure 1:**
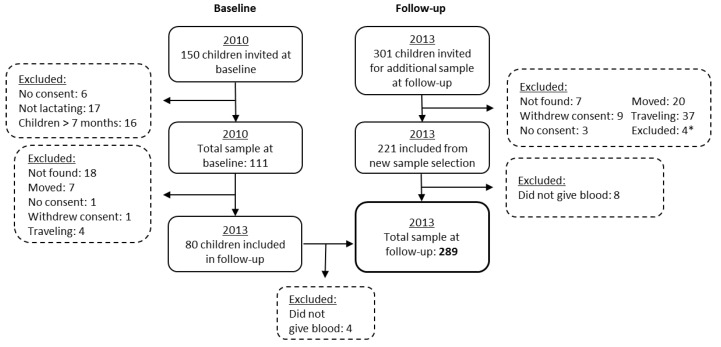
Flowchart for selection of Saharawi children at baseline (2010) and follow-up (2013). * low age, high age, deaf and mute.

The sample size calculations and selection procedures were previously described for the baseline study [21]. Three years later, in 2013, the follow-up study was performed. Among the 111 children from baseline, 76 were included at follow-up. Furthermore, a new sample of randomly selected children from lists made from a census of the Saharawi health authorities (*n* = 213) born between 2010 and 2011 were included to increase the sample size to 289. The sampling procedure and exclusions are described in Figure 1.

### 2.2. Urine and Water Samples

Spot urine samples were collected from all participants at baseline and follow-up. Uri Max urine bags (Servoprax GmbH, Wesel, Germany) were used to collect the children’s urine at baseline. A Vacuette Urine System with transfer device (Vacuette, Krensmünster, Austria) was used for sampling and storage. All samples were stored at −20 °C until analyzed. Analyses of iodine in urine and water were performed by a modified Sandell-Kolthoff reaction [22,23]. Acid digestion by an oxidative reagent, ammonic persulfate, was used to eliminate interfering substances (100 °C for 60 min). Arsenic acid was the reducer reagent in the analyses, and the results were read spectrometrically on microtiter plates. Analyses were performed at the Nutritional Intervention Research Unit in Cape Town, South Africa at baseline and at La Paz University Hospital Foundation for Biomedical Research in Madrid, Spain at follow-up. The World Health Organization (WHO) epidemiological criteria for assessing iodine nutrition based on the median UIC were used to describe the urine data [1].

### 2.3. Breast Milk Samples

Data on BMIC have previously been published with descriptions of the sampling methods and analyses [21]. We estimated the daily iodine intake for children based on BMIC from our study and mean breastmilk intake calculated by the WHO [24] using breastmilk intake in liter * BMIC in µg/L. We used intake data for predominantly breastfed children from developing countries, since these were mostly in accordance with our population.

### 2.4. Anthropometric Measures

Body weight was measured using a UNICEF digital platform scale (SECA 890, Hamburg, Germany). At baseline, the infants’ weight was measured when being held by their mothers after resetting the weight scale. Height/length was measured to the nearest 0.1 cm using a portable UNICEF length board. The gender-specific Z-scores height-for-age (HAZ), weight-for-age (WAZ), and weight-for-height (WHZ) were calculated using the WHO macro for SPSS [25,26]. A child was categorized as undernourished if HAZ, WAZ, or WHZ also referred to as stunting, underweight, and wasting was <−2 [26]. Both at baseline and follow-up, the mothers of the children answered a pre-coded questionnaire concerning the child’s birth date, gender and household size.

### 2.5. Serum Samples and Thyroid Hormones

At follow-up, blood samples were drawn from all participants using plastic clot activator gel tubes (BD Vacutainer SST II, Oxford, UK). Samples were kept at 5 °C until centrifuged and separated from the blood pellet within 2 hours after extraction. Serum samples were stored in transfer tubes and kept frozen at −20 °C until analyzed. Serum thyrotropin (TSH), free thyroxine (fT4), free triiodothyronine (fT3), Tg, and antibodies to thyroid peroxidase (TPOAb) and thyroglobulin (TgAb) were analyzed by electrochemiluminescence immunoassay on Module E170 by Roche Diagnostics at La Paz University Hospital Foundation for Biomedical Research in City, Spain. To classify thyroid hormone disturbances in children, the age-dependent reference range from the manufacturer was used [27]. The reference ranges were 0.70–6.0 mIU/L for TSH, 12.3–22.8 pmol/L for fT4, and 3.7–8.5 pmol/L for fT3. For the thyroid antibodies TgAb and TPOAb, reference values of <38 kIU/L and <13 kIU/L were used, respectively. For Tg, <67 µg/L was used as a reference value. Subclinical hypothyroidism was defined as TSH above the reference and fT4 within the reference. Overt hypothyroidism was defined as TSH above the reference with fT4 below the reference or highly elevated TSH (>10 mIU/L) and fT4 in the lower quartile. Subclinical hyperthyroidism was defined as TSH below the reference and both fT3 and fT4 within the reference. Overt hyperthyroidism was defined with TSH below the reference and fT3 or fT4 above the reference ranges.

### 2.6. Ethical Considerations

Ethical approval for the baseline and follow-up studies (ref. 2010/2513 and 2013/192) was provided by the Regional Committees for Medical and Health Research Ethics in Norway and the Saharawi Ministry of Public Health. Written informed consent was given by the mothers on behalf of the children.

### 2.7. Statistics

Data were analyzed using IBM SPSS version 22 (IBM Corp., Armonk, NY, USA). Normally distributed data were presented as mean (SD), while skewed data was presented with median and 25–75th percentiles (p25–p75) or range.

In linear regression analyses, we measured the associations between different baseline and follow-up variables. We used a purposeful manual forward selection procedure to identify the predictor variables (Table 1). The dependent variables UIC baseline, UIC follow-up, TSH, Tg, and fT3 were log(2) transformed for better fit to the model. The predictor variable Tg was log(2) transformed in the fT4 model due to a non-linear relation. Outliers resulting in standard residuals outside ±3 were removed from the models. The independent variables were tested for association one by one. Only variables with a significant association (*p* < 0.05) were retained in the multiple models. The variables that remained significant in the multiple models are presented in the results (Table 5). All originally selected variables were re-included one by one in the multiple model to check for missed associations. Results regarding TSH, fT3, and fT4 are presented in Table 5, while results regarding UIC from baseline and follow-up, and Tg is presented in the text.

We also examined the association between subclinical hypothyroidism and HAZ, WAZ, and WHZ using multiple logistic regression models. The models were adjusted for breastfeeding status, and the results are shown in Table 6.

## 3. Results

Background characteristics and iodine status from the baseline and follow-up studies are described in Table 2. The median age of the children was 3.1 months at baseline, while it was 31.4 months at follow-up. At baseline, 16.5%, 13.8%, and 8.2% were underweight, stunted, and wasted, respectively. At follow-up, 11.8% were underweight, 33.2% were stunted, and 3.8% were wasted. All children were breastfed at baseline, and 13.8% were still breastfed at follow-up. The median (p25–p75) UIC were 722 (393–1133) µg/L and 458 (275–1026) µg/L at baseline and follow-up, respectively (*p* = 0.003). The median (p25–p75) BMIC at baseline was 479 (330–702) µg/L.

**Figure 2:**
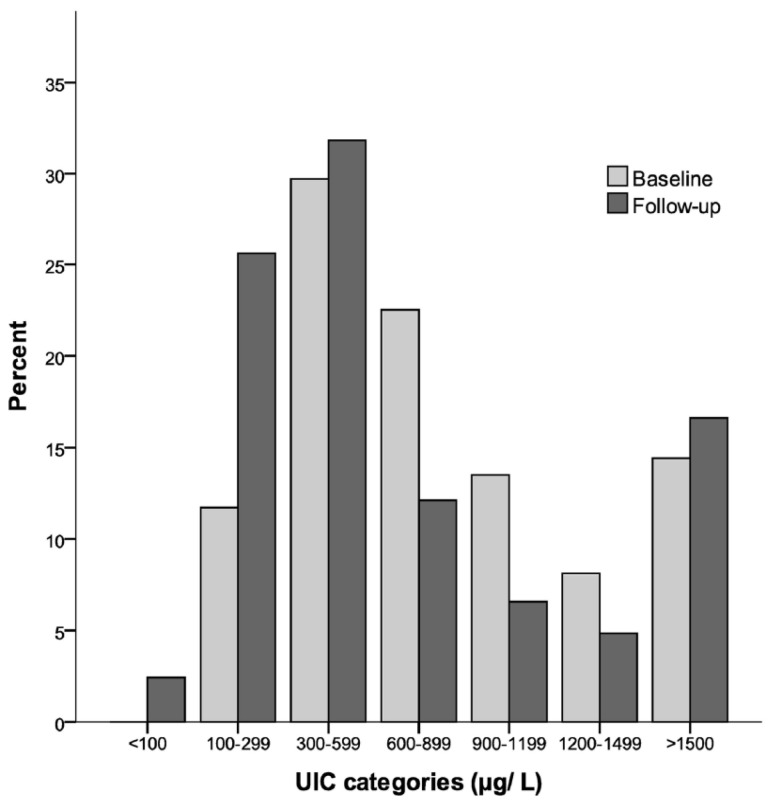
Percentage distribution of children’s UIC at follow-up (*n* = 289) and UIC at baseline (*n* = 111) according to different categories of iodine status.

Figure 2 shows the percentage distributions of children in different categories of iodine nutrition based on the UIC from baseline and follow-up. At baseline, none had a UIC < 100 µg/L, 11.7% had a UIC of 100–299 µg/L, and 88.3% had a UIC ≥ 300 µg/L. At follow-up, 2.4% had a UIC < 100 µg/L, 25.6% had a UIC of 100–299 µg/L, and 71.9% had a UIC ≥ 300 µg/L.

Table 3 shows the daily estimated iodine intake among breastfed children from baseline. Estimated iodine intake from breastmilk increases up to five months of age. Across age groups, the average estimated iodine intake from breast milk is 299 (206–438) µg/day for predominantly breastfed children.

Table 4 describes the serum concentrations of thyroid hormones and Tg, as well as the biochemically assessed thyroid function tests among the children. The median/mean values of thyroid hormones and Tg are within the middle of the reference ranges, except for fT3, which is found in the upper area. No children were found to be TgAb or TPOAb positive (data not shown for thyroid antibodies). We found that 9.3% had subclinical hypothyroidism, and among these, 37.0% had elevated Tg and 3.7% had elevated fT3. None had overt hypothyroidism; however, three children had TSH between 10 and 15 mIU/L with fT4 values above the lower quartile. One child (0.4%) had subclinical hyperthyroidism and one (0.4%) had overt hyperthyroidism. We also found three children (1%) with marginally low fT4 and eight children (2.8%) with elevated fT3 together with TSH within the reference ranges. Two of the children with fT3 and fT4 outside the reference ranges had elevated Tg. In total, thyroid function tests were abnormal among 13.8% of the children. We found that 9.7% had elevated Tg without any thyroid disturbances. In total, 13.2% had elevated Tg, counting those with and without abnormal thyroid function tests. The total prevalence with abnormal thyroid function tests and/or elevated Tg was 23.5%.

Predictors for TSH, fT4, and fT3 at follow-up are shown in Table 5. In the multiple model, Tg and fT3 were positively associated with TSH, explaining 11.3% of the variance in TSH. There were no significant associations between TSH and UIC, fT4, breastfeeding status, age, or gender. fT4 was positively associated with Tg, explaining 1.4% of the variance in fT4. No significant associations were found between fT4 and UIC, breastfeeding status, age, or gender. For fT3, a negative association with UIC was found, where UIC explained 6.7% of the variance in fT3. No significant associations were found between fT3 and Tg, breastfeeding status, age or gender. We also explored predictors for Tg, UIC baseline, and UIC follow-up (data only presented in the text). Tg was associated with TSH as the only variable, explaining 11.6% of the variance. Iodine status, expressed as UIC, was positively associated with BMIC as the only variable at both follow-up and baseline. At baseline, BMIC explained 23.1% of the variance in UIC, while at follow-up, gender was the only significant predictor of UIC, where UIC seemed to be higher among male children than female, explaining 2.9% of the variance.

Nutritional status according to subclinical hypothyroidism is presented in Table 6. Children with subclinical hypothyroidism had significantly lower HAZ (*p* = 0.004) and WAZ (*p* = 0.012) compared to those without hypothyroidism; this result was the same after adjusting for breastfeeding status.

## 4. Discussion

In this study, we found a high prevalence of thyroid disturbances among young children who reside in an area of chronically high iodine exposure. High iodine intakes were identified both at baseline and follow-up.

As illustrated in Figure 2, 88% and 72% had UIC ≥ 300 µg/L at baseline and follow-up, respectively. UIC ≥ 300 µg/L is used as a cut-off by the WHO for indicating iodine excess with a risk of adverse health consequences for school-aged children [1]. However, for younger children, a cut-off indicating excessive iodine intake based on the UIC has not yet been developed. UIC were assessed with spot urine samples, which is the recommended method for assessing iodine status in population groups by the WHO [1], and also recently reviewed as a reliable biomarker for groups [28]. Even so, spot sampling has weaknesses because it is affected by day-to-day hydration level, and there may be wide daily variation in UIC [29,30,31], meaning that an individual showing UIC < 100 µg/L does not necessarily need to be iodine deficient. In this study, all of the children were breastfed at baseline and the mean estimated iodine intake from breast milk was 299 µg/day. This is over three times higher than the recommended daily intake from the WHO of 90 µg/day for children under two years of age [1], and one and a half times as high as the US Institute of Medicine and the European Scientific Committee on Food’s tolerable upper intake level (UL) of 200 µg/day for children of 1–3 years [32,33]. This finding is in accordance with the UIC levels, indicating excessive iodine intakes among the children.

Median UIC was significantly higher at baseline than at follow-up. The iodine content in the urine samples was analyzed in two different laboratories, which may explain some of the differences. Both laboratories used a modified Sandell–Kolthoff reaction for iodine measurement; however, we have no data for comparison analysis. All children from baseline were breastfed, while only a small proportion were breastfed at follow-up, since these children were older. The median BMIC was extremely high (479 µg/L), and since the children were receiving more breastmilk at baseline than at follow-up, the difference in UIC can likely be explained by this.

In the final regression model, BMIC was a predictor of the children’s UIC at baseline. This finding is in concordance with other studies [34,35,36]. Even though the explained variance was small (2.9%), gender was the only predictor of UIC follow-up, whereas male children seemed to have higher UIC than female children, at follow-up. This may be due to different dietary habits between male and female children. However, we do not see gender differences in regards to any of the thyroid parameters. We also explored whether TSH could be associated with iodine status, and TSH was tested for associations with UIC follow-up, UIC baseline, and BMIC at baseline. No significant associations were found for any of the mentioned variables. This may be due to the very large intra-individual variability of the UIC and BMIC measurements. Similarly, no association was found between UIC and TSH in two Iranian studies where urinary iodine was not associated with thyroid function or TSH in newborns in an iodine-sufficient area [35,37], or in a recent study by Nepal et al., where TSH was associated to neither UIC nor serum Tg in children living under chronic iodine excess [15].

Even though neither UIC nor BMIC predicted TSH in our study, others have reported an association between UIC and TSH in children. In a Chinese study of children living under long-term iodine excess, subclinical hypothyroidism was significantly higher among children with UIC > 600 µg/L than among children with a lower UIC [16]. A Korean study among preterm infants found that urinary iodine was significantly higher among children with subclinical hypothyroidism after three weeks, but not after six weeks of life [11]. In our study, serum Tg was positively associated with TSH. Tg has been found to be a sensitive marker of iodine nutrition, also in areas of iodine excess [1,38]. At follow-up, 13.8% of the children were still breastfed. Breastfeeding status was not significantly associated with UIC, TSH, fT4, fT3, or Tg at follow-up.

Evaluating the thyroid hormone status in Table 4, we see that 9.3% had subclinical hypothyroidism. Studies on thyroid function in healthy populations of infants are limited [39,40]. The most cited study on thyroid function in a pediatric population was carried out among US adolescents where 1.7% had subclinical hypothyroidism [41]. A large study among schoolchildren conducted in India after salt iodization revealed prevalences of subclinical and overt hypothyroidism of 6.1% and 0.4%, respectively [42]. However, the results of these two studies may not be comparable to our data, since it is known that thyroid disturbances may increase when improving iodine status after long-term deficiency [43], and because higher age groups were studied than in our material. In a retrospective analysis of TSH from a large database in Israel, elevated TSH was found in 2.9% and highly elevated TSH (>10 mlU/L) in 0.4% of children between six months and 16 years of age [44]. Compared to this study, our prevalence of 9.3% may be considered high, especially taking into account the vulnerable age group of our population group. Studies from other child populations with long-term iodine excess have found similar results to ours; Nepal et al. reported a prevalence of 7.4% with subclinical hypothyroidism and 15.8% with elevated Tg (using the international Tg reference) among Nepalese infants aged 6–24 months [15]. Moreover, Sang et al. found about 5% of Chinese children of 7–13 years had subclinical hypothyroidism [16].

We do not know the optimal reference ranges of thyroid hormones and antibodies in our population. The reference used is of great significance of the reported prevalence of thyroid hormone disturbances. In a population of healthy children aged 2–7 years, the range of TSH was found to be 0.10–5.9 mIU/L, measured by AutoDelfia [45], which corresponds well to the references we have applied.

Ten children (3.5%) had elevated fT3; of which eight had TSH within the reference, one had low TSH, and one had high TSH. All children had normal fT4 values. To the best of our knowledge, the pattern of elevated fT3 in relation to iodine excess has not been described previously. Furthermore, our data suggested a positive association between TSH and fT3 in the multiple regression analyses. This same association was found in a large pediatric population where normal or slightly above normal TSH levels correlated positively with fT3 [46] and not with fT4 among children of normal weight. The authors of the paper stated that the mechanism by which TSH levels are associated with higher fT3 levels and not higher fT4 levels remains unknown.

Studies examining possible outcomes of untreated subclinical hypothyroidism in children remain scarce [39], and there are uncertainties related to the effect of treating the condition among children [47]. Many studies have not seen any clinical manifestations of subclinical hypothyroidism. In our study, children with subclinical hypothyroidism also had statistically significant lower length/height-for-age and weight-for-age than children without, which may suggest a potential link between subclinical hypothyroidism and linear growth and weight faltering, though the observational design of our study limits our ability to infer causality. Improved growth after treatment of subclinical hypothyroidism has been shown in some studies [48,49], and the growth of the children in our study should therefore be monitored.

### Strengths and Limitations

In this study, we have explored an under-reported phenomenon; the occurrence of thyroid dysfunction in a population with excessive iodine exposure. In addition, information on thyroglobulin levels and fT3 levels in children with chronically high iodine exposure is a contribution to the scientific literature on the subject, as there are limited normative data on these analytes. However, our ability to explore associations with iodine status and thyroid function was limited due to limitations of the available biomarkers. Data on thyroid parameters was not collected from baseline because of the very young age of the children.

## 5. Conclusions

In conclusion, we found consistently high iodine intakes among children aged 0–6 months at baseline and three years later at follow-up. The high iodine exposure may have affected thyroid hormone status, possibly resulting in elevated fT3 in some of the children, as well as a higher proportion with elevated TSH and Tg than expected. Iodine excess is an environmental risk factor for the development of thyroid function disorders, especially in susceptible individuals, such as infants, which, in turn, represent a risk factor for developmental delays and poor ponderal and linear growth. Therefore, the thyroid function should be carefully monitored in areas with environmental risk of high iodine exposure, and the need for establishing upper reference levels for iodine nutrition among young children must be addressed. Furthermore, we would recommend purification of ground water in the Saharawi refugee camps, as a strategy to reduce the iodine exposure in the population.

## Figures and Tables

**Table 1:** Overview of predictor variables used in linear regression models for the dependent variables UIC baseline, UIC follow-up, TSH, Tg, fT3 and fT4.

Dependent Variables	Predictor Variables	
	Baseline	Follow-Up
UIC baseline ^b^	HAZ, WAZ, WHZ, age, gender, BMIC	-
UIC follow-up ^b^		HAZ, WAZ, WHZ, breastfeeding status, age, gender
TSH follow-up ^a^		fT3, fT4, UIC, breastfeeding status, age, gender
Tg ^b^		fT3, fT4, UIC, breastfeeding status, age, gender
fT4 ^a^		UIC, Tg, breastfeeding status, age, gender
fT3 ^a^		UIC, Tg, breastfeeding status, age, gender

^a^ Results shown in Table 5 in the results; ^b^ Results presented in the text only. Height/length-for-age (HAZ), Weight-for-age (WAZ), Weight-for-height (WHZ).

**Table 2:** Background characteristics and iodine status among the children at baseline and follow-up ^a^.

Characteristics	Baseline (*n* = 111)	Follow-up (*n* = 289)
Age, *months*	3.1 (2.2–4.8)	31.4 (25.3–35.1)
Male	40 [36.0]	138 [47.8]
Female	71 [64.0]	151 [52.2]
Household size, *number*	5.1 ± 1.9	5.3 ± 1.8
Breast-fed	111 [100.0]	40 [13.8]
Weight-for-age, *z-score*	−0.8 ± 1.3	−1.0 ± 0.9
<−2 (underweight)	18 [16.5]	34 [11.8]
Length/height-for-age, *z-score*	−0.6 ± 1.2	−1.6 ± 1.1
<−2 (stunted)	15 [13.8]	96 [33.2]
Weight-for-length/height, *z-score*	−0.3 ± 1.2	−0.1 ± 1.0
<−2 (wasted)	9 [8.2]	11 [3.8]
Iodine status		
UIC, *µg/L*	722 (393−1133)	458 (275−1026) *
BMIC, *µg/L* ^b^	479 (330−702)	

^a^ Values are presented as mean ± SD, median (p25–p75), and n [%]. Two missing from anthropometric data baseline, one from follow-up. One missing UIC follow-up. One missing age child baseline; ^b^ Data for BMIC is previously published by Aakre et al. [20,21]. Only measured at baseline. * UIC baseline and follow-up were statistically significant different (*p* = 0.003) tested with Mann–Whitney U.

**Table 3:** Estimated iodine intake in age groups 1–6 months based on median (p25–p75) BMIC.

Age in Months	Estimated Breast Milk Intake (g/Day) (3)	Estimated Iodine Intake from Breast Milk (µg/Day) ^a^
1	568	272 (187–399)
2	636	305 (210–446)
3	574	275 (189–403)
4	643	308 (212–451)
5	714	342 (236–501)
6	611	293 (202–429)
Mean	624	299 (206–438)

^a^ Calculated by breastmilk intake (3) in g/day*mean (p25–p75) BMIC in µg/g for our study.

**Table 4:** Thyroid hormone status and thyroid function tests among children at follow-up ^a^.

Thyroid Hormones and Thyroid Function Tests	Children (*n* = 289)	Reference Range
TSH, *mIU/L*	3.1 (0.2–15.3)	0.70–6.0
fT4, *pmol/L*	16.3 (11.2–21.8)	12.3–22.8
fT3, *pmol/L*	6.9 (3.9–17.2)	3.7–8.5
Tg, *µg/L*	38.4 (10.7–158.0)	<67
Subclinical hypothyroidism ^b^	27 [9.3]	
Subclinical hyperthyroidism	1 [0.4]	
Overt hyperthyroidism	1 [0.4]	
fT4 low <12 pmol/L, normal TSH ^c^	3 [1.0]	
fT3 elevated >8.5 pmol/L, normal TSH ^d^	8 [2.8]	
Tg elevated >67 µg/L, normal thyroid tests	28 [9.7]	
Total thyroid disturbance and/or elevated Tg	68 [23.5]	

^a^ Data are presented as median/mean (min-max) and *n* [%]; ^b^ Among children with subclinical hypothyroidism there were 10 [37%] with elevated Tg, one had elevated fT3; ^c^ Among low fT4, one had elevated Tg; ^d^ Among children with elevated fT3, one had elevated Tg.

**Table 5:** Predictors for TSH, fT4 and fT3 (*n* = 289).

Dependent Variables ^a^	Predictor Variables	Unadjusted/Adjusted Coefficient (95% CI) ^d^	*p*	Stand Beta	*R*^2^
TSH	Tg *mg/L*	6.2 (3.9−8.5)	*<0.001*	0.297	
	fT3 *pmol/L*	0.09 (0.02−0.15)	*0.011*	0.143	0.113
fT4 ^b^	Tg ^c^	0.3 (0.01, 0.5)	*0.043*	0.120	0.014
fT3	UIC follow-up *mg/L*	−0.04 (−0.06, −0.02)	*<0.001*	−0.260	0.067

^a^ The dependent variables TSH and fT3 were log (2) transformed, *n* = 286 for TSH, *n* = 284 for fT4 and *n* = 283 for fT3; ^b^ Ft4 is given in pmol/L; ^c^ Tg as a predictor variable was log(2) transformed due to a non-linear relation; ^d^ TSH model adjusted for Tg and fT3. In fT4 and fT3 models, unadjusted coefficients are shown.

**Table 6:** Nutrition status among children with subclinical hypothyroidism at follow-up ^a^.

Nutrition Status	Subclinical Hypothyroidism		*p*
	Yes (*n* = 27)	No (*n* = 260) ^b^	
Length/height-for-age, *z-score*	−2.2 ± 1.0	−1.6 ± 1.1	*0.004*
Weight-for-age, *z-score*	−1.4 ± 0.8	−0.9 ± 1.0	*0.012*
Weight-for-length/height, *z-score*	−0.3 ± 0.8	−0.1 ± 1.0	*0.349*

^a^ Values are presented as mean ± SD. Differences are tested by independent sample *t*-tests. Associations between HAZ, WAZ and TSH levels remained significant when adjusted for breastfeeding status in multiple regression analyses; ^b^ Two children with hyperthyroidism were excluded from the analyses.
